# Serine/Threonine Kinase 3-Phosphoinositide-Dependent Protein Kinase-1 (PDK1) as a Key Regulator of Cell Migration and Cancer Dissemination

**DOI:** 10.3390/cancers9030025

**Published:** 2017-03-11

**Authors:** Laura Di Blasio, Paolo A. Gagliardi, Alberto Puliafito, Luca Primo

**Affiliations:** 1 Candiolo Cancer Institute FPO-IRCCS, 10060 Candiolo, Torino, Italy; laura.diblasio@ircc.it (L.D.B.); paolo.gagliardi@ircc.it (P.A.G.); alberto.puliafito@ircc.it (A.P.); 2 Department of Oncology, University of Torino, 10043 Orbassano, Torino, Italy

**Keywords:** 3-phosphoinositide dependent protein kinase-1 (PDK1), phosphatidylinositol-3-kinase (PI3K), cell migration, cancer

## Abstract

Dissecting the cellular signaling that governs the motility of eukaryotic cells is one of the fundamental tasks of modern cell biology, not only because of the large number of physiological processes in which cell migration is crucial, but even more so because of the pathological ones, in particular tumor invasion and metastasis. Cell migration requires the coordination of at least four major processes: polarization of intracellular signaling, regulation of the actin cytoskeleton and membrane extension, focal adhesion and integrin signaling and contractile forces generation and rear retraction. Among the molecular components involved in the regulation of locomotion, the phosphatidylinositol-3-kinase (PI3K) pathway has been shown to exert fundamental role. A pivotal node of such pathway is represented by the serine/threonine kinase 3-phosphoinositide-dependent protein kinase-1 (PDPK1 or PDK1). PDK1, and the majority of its substrates, belong to the AGC family of kinases (related to c**A**MP-dependent protein kinase 1, cyclic **G**uanosine monophosphate-dependent protein kinase and protein kinase **C**), and control a plethora of cellular processes, downstream either to PI3K or to other pathways, such as RAS GTPase-MAPK (mitogen-activated protein kinase). Interestingly, PDK1 has been demonstrated to be crucial for the regulation of each step of cell migration, by activating several proteins such as protein kinase B/Akt (PKB/Akt), myotonic dystrophy-related CDC42-binding kinases alpha (MRCKα), Rho associated coiled-coil containing protein kinase 1 (ROCK1), phospholipase C gamma 1 (PLCγ1) and β3 integrin. Moreover, PDK1 regulates cancer cell invasion as well, thus representing a possible target to prevent cancer metastasis in human patients. The aim of this review is to summarize the various mechanisms by which PDK1 controls the cell migration process, from cell polarization to actin cytoskeleton and focal adhesion regulation, and finally, to discuss the evidence supporting a role for PDK1 in cancer cell invasion and dissemination.

## 1. Introduction

### 1.1. Cell Migration

Cell migration is a fundamental process both in physiological situations (such as embryonic development, inflammatory response and wound healing) and in pathological ones (tumor progression and angiogenesis, osteoporosis and chronic inflammatory disease) [1]. Cell locomotion is regulated by a complex network of signaling events that involves lipid second messengers, kinases, small GTPases and cytoskeletal proteins.

Cell migration can be described prototypically as a cyclic process [2]. The first step is the polarization of the cell in response to migration-promoting factors. As a consequence, the cell extends different protrusions, either in the form of large lamellipodia or finger-like filopodia, driven by actin polymerization. Subsequently, cells establish new integrin-mediated adhesions with the underlying substrate in correspondence of protrusions; these nascent adhesions, linked to the actin cytoskeleton, will mature and provide a traction site to the cell to retract its rear by means of myosin II contraction.

These different steps can be observed, albeit with peculiarities, in a range of different cell types, both epithelial and mesenchymal, and in different environments in response to various chemoattractants.

The mesenchymal migration mode is predominantly used by cells originating from connective-tissue tumors, such as fibrosarcomas, gliomas, and from epithelial cancer tissues. Carcinoma cells crawling on extracellular matrix (ECM) fibers extend pseudopods functionally equivalent to lamellipodia [3]. Integrins, MT-MMPs (membrane-type matrix metalloproteinases) and other proteases colocalize at the edge of pseudopods to contribute to pericellular proteolysis [4]. Instead, many other tumor cells use a less adhesive, amoeboid mode of migration [5]. Amoeboid motility has been mainly studied in *Dictyostelium discoideum*, while in higher eukaryotes this migration mode is characteristic of lymphocytes and neutrophils [6]. Cells migrating in this fashion move fast by gliding on the substrate, only supported by cortical filamentous actin and contraction and without the need of both focal adhesions and proteolysis. 

Cells can move as cell strands/sheets/clusters as well (collective migration). In physiological situations, this mode of migration can occur during embryonic development, morphogenesis of mammary glands and ducts and sprouting angiogenesis [4], but this mode of migration can be found in tumor cells as well. Notably, cancer cells can change their molecular migration program and undergo a variety of transitions between the different migration modes (such as epithelial–mesenchymal transition or mesenchymal–amoeboid transition) [7].

### 1.2. PI3K

**Figure 1:**
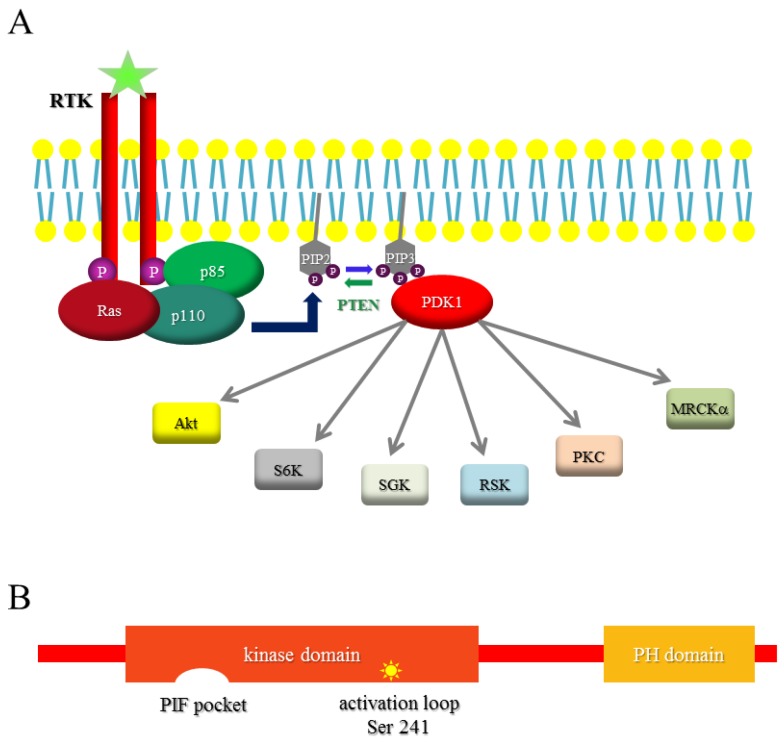
The phosphatidylinositol-3-kinase (PI3K)–3-phosphoinositide-dependent protein kinase-1 (PDK1) pathway. (**A**) Schematic representation of the pathway activated by PI3K through PDK1. Receptor-stimulated class I PI3Ks generate phosphatidylinositol (3,4,5) trisphosphate (PIP3), which bind directly to the pleckstrin homology domain of PDK1, which in turn activates a plethora of downstream targets, a selection of which is shown, with different mechanisms (kinase-dependent or -independent; pleckstrin homology (PH) domain-dependent, etc.); (**B**) PDK1 structure. PDK1 contains an N-terminal kinase domain and a C-terminal PH domain. Inside the kinase domain, there are two important sites: the PDK1 interacting fragment (PIF)-pocket and the activation loop; the latter comprises serine 241, which is essential for PDK1 kinase activity and is constitutively phosphorylated.

Among the pathways involved in the regulation of cell migration, the phosphatidylinositol-3-kinase (PI3K) pathway has been shown to be fundamental. PI3Ks are important for maintenance of polarity and definition of the leading edge of the cell, as well as for effective migration [8]. PI3K lipid kinases are grouped into three distinct classes on the basis of their substrate specificity and sequence homology: class I (A and B), class II and class III [9]. PI3Ks generate lipid second messengers by phosphorylating the head group of membrane-anchored phosphoinositides at the 3′ position, which bind and regulate downstream protein effectors containing the pleckstrin homology (PH) domain. Classes IA and IB, together with their lipid product phosphatidylinositol (3,4,5) triphosphate (PIP3), are widely implicated in controlling cell migration and polarity. The PI3K signaling cascade is mainly mediated by the activation of the serine/threonine kinases of the AGC (related to cAMP-dependent protein kinase 1, cyclic Guanosine monophosphate-dependent protein kinase and protein kinase C) family, such as 3-phosphoinositide-dependent protein kinase-1 (PDPK1 or PDK1), protein kinase B/Akt (PKB/Akt), p70S6K, serum- and glucocorticoid-dependent protein kinase (SGK), and p90 ribosomal protein S6 kinase (p90RSK) [10,11] (Figure 1A). Besides Akt and PDK1, other key effectors of PI3K in the regulation of migration process are for example GDP–GTP exchange factors (GEF) for Rac and for ADP-ribosylation factors 6 (ARF6) and GTPase activating proteins (GAP) of Rho GTPases [12]. 

### 1.3. PDK1

A crucial node of the PI3K pathway is represented by the serine/threonine kinase PDK1). PDK1 was discovered in 1997 as the kinase responsible for the phosphorylation of Akt on the activation loop, at threonine 308, which is essential for Akt activation [13]. PDK1 is a protein of 556 amino acids with an N-terminal catalytic domain and a C-terminal pleckstrin homology (PH) domain (Figure 1B). Similar to other AGC kinases, PDK1 contains a phosphorylation site within the activation loop (serine 241), which is constitutively phosphorylated by an autophosphorylation reaction in *trans* [14]. PDK1 kinase is therefore considered constitutively active.

The regulation of PDK1-activated signaling is based on different mechanisms [15]. The first mechanism is depicted by phosphorylation of Akt activation loop. PDK1 localizes at the plasma membrane due to the interaction of its PH domain with PIP3 (and to a lesser extent with phosphatidylinositol (3,4) bisphosphate) produced by PI3K and thus physically interacts with and phosphorylates Akt [16]. The second mechanism of activation for substrates lacking a PH domain (p70S6K, SGK, p90RSK and PKC isoforms) is PIP3-independent. On the kinase domain, PDK1 possesses a hydrophobic pocket, termed the PDK1 interacting fragment (PIF) pocket, which allows its interaction with the phosphorylated hydrophobic motif of the targeted kinases and the consequent phosphorylation of their activation loop [17,18,19]. Moreover, PDK1 activity is also regulated by reversible tyrosine phosphorylation [20]. Three tyrosine phosphorylation sites have been identified, tyrosine 9, 373 and 376, but only phosphorylation on tyrosines 373/376 is important for PDK1 activity. Src tyrosine kinase can phosphorylate all the three sites [20,21], while Pyk2 can phosphorylate only tyrosine 9 [22].

The physiological role of PDK1 has been extensively investigated in vivo in murine models (see Table 1 for a summary of different conditional knockout models). Knockout of PDK1 is lethal, indicating its requirement for normal embryo development [23]. PDK1 knockout mice die at the E9.5 embryonic stage, showing lack of branchial arches, defects in neural crest-derived tissues and forebrain development, as well as defective assembly of a functional vascular system. To understand the role of PDK1 during development, hypomorphic mice for PDK1 have been generated, in which the expression of PDK1 is reduced by 80%–90% in all tissues. These mice are viable and show a decreased body size, but no significant differences in the activation of Akt, p70S6K, and p90RSK.

Notably, some of the defects found during development of knockout embryos might be due to deficient migration. Actually, PDK1 has been demonstrated to regulate cell migration in multiple ways [24]. Here we aim at summarizing how PDK1 controls cell migration at different levels, from cell polarization to actin cytoskeleton and focal adhesion regulation.

## 2. Polarization of Signaling

To execute persistent migration, cells establish leading and trailing edges in which different signaling pathways stimulate membrane protrusion and retraction, respectively. In most cases, cell orientation is determined by external gradients of soluble and/or adhesive factors. Even in the absence of such cues, persistence and internal spatial organization of intracellular signaling can still be observed and is correlated with bias in the direction of migration. The maintenance and/or dynamic changes of cell polarity are governed by asymmetric spatial distribution and activation of intracellular signaling proteins. In the presence of external concentration gradients of chemoattractants, receptors are locally activated in a measure proportional to the local amount of available ligand. This, often small, difference in activated receptors is then amplified by a signaling network and translates into a bias in the direction of cell migration. Such general view, often referred to as “gradient sensing”, attempts to explain the ability of cells to generate amplified, persistent intracellular signaling to static, external gradients of chemoattractants, as well as transient responses to uniform stimuli. Many of the models that have been proposed to explain gradient sensing are based on a local excitation, global inhibition (LEGI) principle [42,43,44]. After receptor stimulation, a fast, local excitatory signal as well as a slower, global, inhibitory (typically thought as generated by a diffusible molecule) signal are activated, causing the polarization of signaling necessary for cell migration. The LEGI model explains the gradient sensing response of most of the molecules that have been shown to move to or be activated at the front (e.g., PI3K, PH domain and actin binding proteins, RAS GTPase) or rear (e.g., phosphatase and tensin homolog [PTEN], myosin). Generally, such models cannot explain the details of cell polarization. Models taking into account such aspects typically include positive feedback loops [45], to reinforce and amplify the gradient sensing response. The positive feedback also helps to explain how polarized cells acquire and maintain a distinct morphology at their front and back. 

The preferential activation of PI3K at the leading edge during directional movement has been studied in *Dictyostelium discoideum* and in leukocytes [42,46,47]. While the chemoattractant receptors (for these cells, G-protein-coupled receptors, GPCRs) are uniformly distributed along the plasma membrane and the G proteins show a very shallow anterior-posterior gradient [48,49,50], proteins carrying PH domains rapidly and transiently translocate to the plasma membrane in response to uniform chemoattractant stimulation [51,52]. More importantly, in chemotaxing cells, these proteins localize to the leading edge. These data provided the first evidence that a marked PIP3 polarization is produced along the membrane of chemotaxing cells in response to a shallow chemoattractant gradient. The persistence of the PIP3 distribution is guaranteed by the tumor suppressor PTEN. PTEN is a phosphoinositide 3′-specific phosphatase that dephosphorylates phosphatidylinositol (3,4,5) triphosphate and phosphatidylinositol (3,4) bisphosphate [53]. In chemotaxing *Dictyostelium*, PTEN is excluded from the leading edge but localizes at the sides and the back of the cell to allow the accumulation of PIP3 only at the front of the cell. Thus, PI3K and PTEN show opposite patterns of spatial localization [54,55].

**Figure 2:**
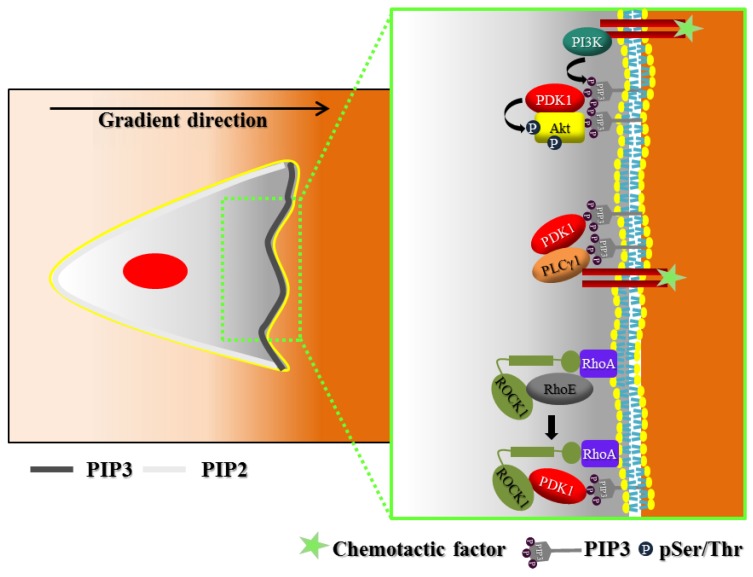
PDK1 contributes to polarization of signaling downstream to PI3K during cell migration. In the presence of a gradient of chemoattractant, a migrating cell is able to polarize following the direction of the gradient. This polarization is achieved by the localized activation of signaling proteins either at the front or at the rear of the cell. The PI3K pathway is activated at the leading edge of migrating cells, with the consequent accumulation of PIP3 (dark grey line). Conversely, while PI3K is excluded from the sides and the back of moving cells, the phosphatase PTEN specifically localizes to such portions of the cell, causing the accumulation of PIP2 (light grey line). The green box shows a detail of signaling activated by PDK1 at the leading edge, downstream to PI3K. First, PDK1 phosphorylates and activates Akt at front of migrating cells. Moreover, through a kinase-independent mechanism, PDK1 is able to stimulate function of phospholipase C gamma 1 (PLCγ1) and ROCK1.

PDK1 has been shown to contribute to the establishment of cell polarity downstream to PI3K (Figure 2). Indeed, by binding PIP3 with its PH domain, PDK1 is able to locally activate a series of PI3K pathways effectors at the leading edge of migrating cells. Primarily, PDK1 activates Akt at the front of moving cells [56,57,58]. In particular, it has been demonstrated that PDK1 overexpression increases vascular endothelial growth factor-A (VEGF-A)-induced cell migration, while PDK1 knockout completely blocks migration capacity of embryoid bodies-derived endothelial cells. Moreover, VEGF-A stimulation induces accumulation of PIP3 at the front of migrating endothelial cells and consequently translocation of both PDK1 and Akt at the leading edge, where PDK1 phosphorylates and activates Akt [56]. In addition, the PDK1–Akt axis regulates chemotaxis of MDA-MB-231 cancer cells toward epidermal growth factor (EGF) [57] and of T-cells [58]; the same axis regulates neocortical neurons locomotion in developing mammalian neocortex [59]. 

PIP3 is essential for the localization to plasma membrane of other two effectors of PDK1, ROCK1 and MRCKα [60,61]. ROCK1 and MRCKα belong to the AGC kinase family and are effectors of small GTPases RhoA (Ras homolog gene family, member A) and CDC42 (cell division control protein 42 homolog), respectively [10]. Both proteins regulate myosin contraction by the phosphorylating myosin regulatory light chain 2 (MLC2) and the myosin phosphatase target subunit 1 (MyPT1) [62,63]. PDK1’s PIF pocket directly interacts with hydrophobic motif of both ROCK1 and MRCKα and guides both proteins to the plasma membrane by means of its PH domain. Furthermore, PDK1 interaction with ROCK1 and MRCKα increases their kinase activity. For ROCK1, the mechanism involves its negative regulator RhoE, since PDK1 competes with RhoE for the binding with ROCK1. ROCK1 activated by PDK1 regulates amoeboid-type cancer cell invasion [60]. Conversely, the activation of MRCKα by PDK1 controls epithelial cell migration and collective invasion [61].

Recently, it has been reported that PDK1 regulates cell migration also through phospholipase C gamma 1 (PLCγ1) [64]. PLCγ1 hydrolyzes phosphatidylinositol (4,5) bisphosphate into diacylglycerol and inositol (1,4,5) trisphosphate (Ins3P) [65]. After growth factor stimulation, PLCγ1 and PDK1 dynamically associate at the plasma membrane through their binding to PIP3 [66]. Moreover, PDK1 downregulation causes decreased PLCγ1 phosphorylation on tyrosine 783.

## 3. Actin Cytoskeleton Regulation

The principal consequence of polarization is the extension of active membrane protrusions, including lamellipodia and filopodia at the cell front. Lamellipodia are large, flat, sheet-like structures, whereas filopodia are thin, cylindrical, finger-like formations [2]. Extension of both lamellipodia and filopodia in response to chemoattractants is coupled with local actin polymerization. Depending on the type of protrusion, actin filaments are differently organized: in lamellipodia, actin filaments form a branching network, whereas in filopodia they are organized into long parallel bundles [67]. Small GTPases of the Rho family and their effectors are pivotal regulators of actin organization and thus of lamellipodia and filopodia formation. Many effectors are activated by Rho GTPases to organize the actin cytoskeleton during cell migration [68]. For example, Cdc42 activates WASp and N-WASp, while Rac activates the Scar/WAVE family. Members of the WASp/SCAR/WAVE family of proteins are key regulators of actin polymerization, because they are able to stimulate the Arp2/3 complex [69]. The Arp2/3 complex induces the formation of a new daughter filament from a preexisting one, thus controlling extension of lamellipodia [70]. An important downstream target of Rho for regulating actin assembly is mDia, which belongs to the formin family of proteins. Furthermore, several actin-binding proteins regulate actin polymerization in protrusions by affecting the pool of available G-actin monomers and free ends [71]. In addition, disassembly of older filaments is controlled by proteins of the ADF/cofilin family, which sever filaments and promote actin dissociation from the pointed end. Filopodia extension occurs through a treadmilling mechanism, in which actin filaments within a bundle elongate at their barbed ends and lose actin monomers from their pointed ends [67]. Proteins enriched in filopodia include Ena/VASP, which bind barbed ends, and fascin, which bundles actin filaments.

**Figure 3:**
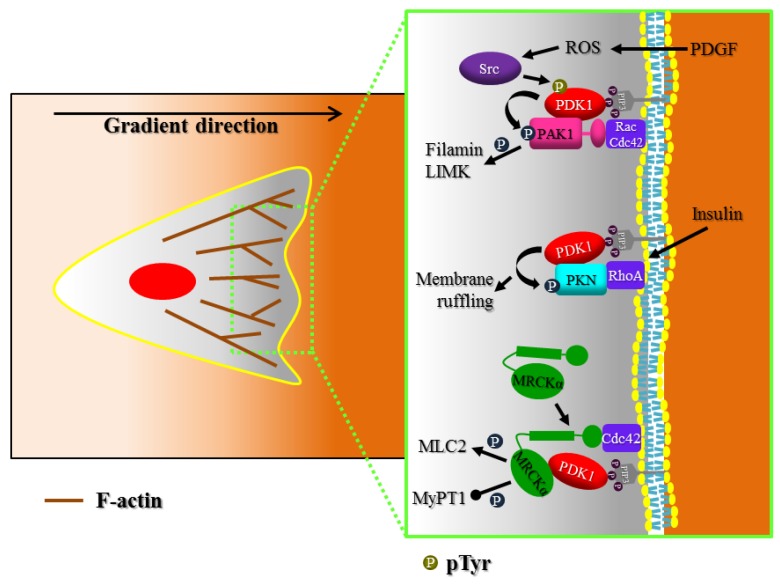
PDK1 regulates membrane protrusions and actin polymerization. After being polarized, migrating cells have to extend active membrane protrusions, including lamellipodia and filopodia at the cell front. Extension of both lamellipodia and filopodia in response to chemoattractant is almost universally found coupled with local actin polymerization. PDK1 controls this process through the phosphorylation of p21-activated kinase 1 (PAK1) and protein kinase N (PKN), downstream to both PI3K and Rho GTPases. On the contrary, PDK1 regulates activity of MRCKα through a kinase-independent mechanism.

PDK1 has been shown to regulate lamellipodial dynamics through MRCKα [61] (Figure 3). In response to chemoattractant stimulation, MCF10A cells exhibit a phase of increasing spreading by lamellipodia extension; then a phase of lamellipodial retraction follows. Both PDK1 and MRCKα dynamically localize at the plasma membrane of extending lamellipodia, but only the retraction phase is totally regulated by the PDK1-mediated regulation of MRCKα. Indeed, when PDK1 is overexpressed both protrusion and retraction phases induced by EGF are modified, while MRCKα silencing blocks only the promoting effect of PDK1 overexpression on retraction phase.

Moreover, PDK1 controls protrusions dynamics by activating p21-activated kinase 1 (PAK1). PAK1 is a serine/threonine kinase that regulates cytoskeletal dynamics mainly downstream to Cdc42 and Rac1 [72]. However, PAK1 activity can also be regulated by different mechanisms including PDK1 phosphorylation at threonine 423 [73]. Upon activation, PAK1 localizes to the leading edges of motile cells and stimulates both motility and invasion [74]. PDK1 and PAK1 regulate vascular smooth muscle cell (VSMC) migration toward platelet-derived growth factor (PDGF) [21]. VSMC, stimulated with PDGF, accumulates reactive oxygen species (ROS), which determine the activation of Src. Then Src phosphorylates PDK1, which in turn phosphorylates and activates PAK1.

Furthermore, PDK1 may regulate actin cytoskeleton through the Rho-activated serine/threonine protein kinase N (PKN) [75]. It has been shown that PKN interacts with PDK1 in vitro and is phosphorylated and activated by PDK1 in cells. Overexpression of PKN or PDK1 induces actin cytoskeleton reorganization (actin stress fiber depolymerization and membrane ruffling) while expression of mutant forms of either PKN or PDK1 inhibits insulin-induced actin cytoskeleton remodelling. These data indicate that phosphorylation of PKN by PDK1 is important to mediate regulation of the actin cytoskeleton by insulin.

## 4. Focal Adhesion and Integrin Signaling

For mesenchymal and epithelial migration to occur, the actin-rich protrusions, which contain several receptors for extracellular matrix proteins, must bind to the substratum. Integrins are the major family of receptors for adhesive molecules of the extracellular matrix (ECM) and play key roles in development, immune responses, leukocyte traffic, angiogenesis and cancer [76]. Integrins basically connect ECM with the actin cytoskeleton inside the cell and activate many migration-related signaling molecules (“outside-in signaling”). They are also transducers of “inside-out signaling”, that is, activation to a high affinity state by cytoplasmic signals [77]. Integrins are heterodimeric receptors consisting of α and β subunits, with large ligand-binding extracellular domains and short cytoplasmic domains [78]. The binding to molecules of the ECM leads to conformational changes in the extracellular domain and to integrin clustering. This combination of binding and clustering initiates intracellular signals that regulate the formation of adhesion sites. Activated integrins preferentially localize to the leading edge of migrating cells, where new adhesions form [79]. Adhesions assemble as small clusters of integrins, known as focal complexes, which stabilize the lamellipodium, and then eventually mature in more stable focal adhesions (FA) or turn over [80,81]. At the rear of a migrating cell, FAs may be disassembled or left on the substratum [82,83]. Microtubules control FA disassembly either through the regulation of Rho GTPase [84] or through a FAK/dynamin pathway [85]. Clathrin and some of its adaptors (e.g., AP-2 and Dab2) are also involved in this process, by mediating integrin endocytosis from disassembling adhesion sites [86,87].

**Figure 4:**
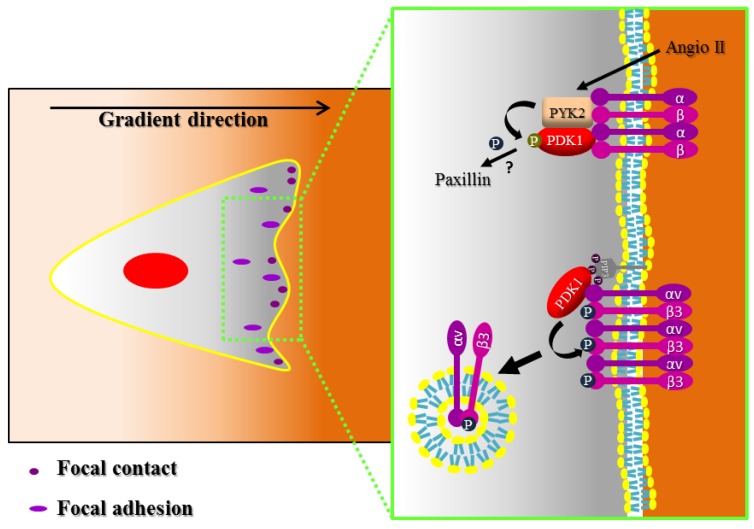
PDK1 regulates focal adhesion and integrin signaling. For migration to occur, the protrusions must stabilize by attaching to the substratum through integrin-mediated adhesions. Adhesions assemble as small clusters of integrins, known as focal complexes, which stabilize the lamellipodium, and then eventually mature in more stable focal adhesions or turn over. PDK1 has been shown to localize to focal adhesions together with Pyk2 and to regulate them, possibly by phosphorylating effectors such as paxillin, through an unknown mechanism. Moreover, downstream to PI3K, PDK1 regulates focal adhesion disassembly, by phosphorylating integrin β3 and thus by inducing its endocytosis. ? refers to unknown mechanism of phosphorylation

Evidence of a PDK1 role in the regulation of adhesions is present in the literature [22,88] (Figure 4). In the first study, it has been shown that both Pyk2 and tyrosine-phosphorylated PDK1 localize in FAs in VSMC after angiotensin II stimulation. Moreover, the tyrosine phosphorylation of PDK1 by Pyk2 is essential for the formation of FA, possibly through downstream regulation of paxillin phosphorylation. Indeed, expression of a PDK1 mutant in one tyrosine phosphorylated by Pyk2 (Y9F PDK1) impaired FA formation by angiotensin II. Moreover, angiotensin II-induced phosphorylation of paxillin is significantly inhibited by Y9F PDK1 [22].

In the second study, it has been shown that PDK1 regulates β3 integrin endocytosis and thus FA disassembly in endothelial cells [88]. Integrin αvβ3 is particularly important in the vascular system as receptor of RGD (Arg-Gly-Asp)-containing ECM proteins (vitronectin and fibronectin) [89]. Interestingly, when PDK1 is downregulated, FA disassembly slows down and FA increase in number and size. This phenotype is the result of the altered endocytosis of integrin αvβ3. Kirk et al. have shown that PDK1 and Akt phosphorylate in vitro the β3 integrin cytoplasmic tail on threonine 753 [90]. The phosphorylation of this residue blocks recruitment of Shc, suggesting that threonine phosphorylation of β3 may be an important modulator of integrin function. PDK1 is responsible for the phosphorylation of threonine 753 of β3 also in vivo in endothelial cells and the mutation to alanine of this residue reduces the internalization of β3 integrin. Beside the PDK1 kinase activity, β3 integrin endocytosis and FA dynamics require also the PDK1 binding to PIP3, downstream to PI3K activation.

## 5. Tumor Invasiveness and Dissemination

The first study showing that PDK1 expression confers not only a growth advantage, but also an invasive phenotype, has been carried out in mammary epithelial cells. Glazer et al. describe an increase of MMP-2 activity and MT1-MMP expression in PDK1-expressing cells, resulting in enhanced invasion on Matrigel [91]. The role of PDK1 in controlling metalloprotease activity was later confirmed by its involvement in invadopodia formation [92]. Invadopodia are adhesive and degradative structures that were initially observed in vitro as shallow protrusions on the baso-lateral side of cultured cancer cells [93]. The ability to form invadopodia is closely related to invasive and metastatic properties in vivo [94,95]. Invadopodia-like protrusions in breast cancer cells have been observed during intravasation by intravital imaging [96], and recently, direct evidence of a functional role for invadopodia during cancer cell extravasation and distant metastasis has been provided [97]. Notably, the expression of an active p110α catalytic subunit (PIK3CA) of PI3K promoted invadopodia-mediated invasive activity, which was blocked by knockdown or inhibition of PDK1 [92].

In a genetic mouse model of melanoma driven by melanocyte-specific expression of BrafV600E and inactivation of PTEN, the genetic inactivation of PDK1 delays the onset of the disease and almost completely abolishes metastases [98]. In the same model, treatment with PDK1 inhibitors effectively reduces melanomagenesis and metastatic load, phenocopying the genetic inactivation.

Expression of KRAS^G12D^ or KRAS^G12V^ in the murine pancreas gives rise to lesions called pancreatic intraepithelial neoplasia (PanIN) that progress to metastatic pancreatic ductal adenocarcinoma (PDAC). In this murine model of pancreatic cancer PDK1 has been found to play an important role in both pancreatic cancer initiation and progression [99]. Indeed, PDK1 knockout in epithelial compartment of the pancreas completely blocks PanIN and PDAC formation. In contrast, deletion of PDK1 in a KRAS^G12D^-driven non-small-cell lung carcinoma (NSCLC) model has no effect on lung tumor formation.

A microRNA-mediated regulation of PDK1 has been described in gastric cancer cells, where miR-128b targets PDK1 thus decreasing cell viability and inhibiting invasion; this effect is achieved through the inactivation of the Akt/NF-κB axis [100]. In all these instances, the role of PDK1 is mainly mediated by Akt. However, accumulating data show Akt-independent effects in cellular models of PDK1 overexpression in term of both growth and invasiveness. In PIK3CA mutant cancer cell lines and in human breast tumors, PDK1 may activate an alternative signal that engages downstream substrates such as SGK3. Thereby, both PDK1 and SGK3 are considered as key oncogenic effectors downstream of activating PIK3CA mutations [101]. However, PDK1 has been reported to regulate breast cancer growth in Akt-independent manner also in absence of PIK3CA mutations [102].

Notably, in colon cancer cells, PDK1 deletion impairs the ability of these cells to form liver metastasis after injection into spleen of immunodeficient mice [103]. Although this effect can be also obtained by the combined deficiency of AKT1 and AKT2, different signaling pathways are activated in PDK1 or AKT1/2 KO cells. The phosphorylation of both mTOR and GSK3β is significantly reduced only in PDK1 KO cells, suggesting the existence of parallel pathways activated by PDK1.

Furthermore, as described in detail above, PDK1 has been described to regulate migration and invasion through a kinase-independent mechanism by activating ROCK1 and MRCKα [60,61]. The PDK1-mediated activation of ROCK1 has been shown to be relevant for amoeboid-type of cell invasion. During amoeboid invasion, PDK1 regulates cortical acto-myosin and is responsible for the movement in collagen/Matrigel matrix [60].

In contrast, the activation of MRCKα by PDK1 is more important for the migration and invasion of epithelial cells. MRCKα regulates directional migration of epithelial cells and collective migration in a three-dimensional environment by controlling lamellipodia dynamics [61].

A different Akt-independent mechanism involves PLCγ1. It has been reported that PDK1 regulates EGF-induced PLCγ1 activation, specifically at the level of cell protrusions, and modulation of PLCγ1 tyrosine phosphorylation. The interaction PDK1–PLCγ1 is important for cancer cell invasion, in particular of breast cancer and melanoma cells [64]. Interestingly, the same group demonstrated that the inositol-1,3,4,5,6-pentakisphosphate derivative, 2-*O*-benzyl-*myo*-inositol 1,3,4,5,6-pentakisphosphate (2-*O*-Bn-InsP_5_), prevented the formation of this PDK1–PLCγ1 complex by binding to the PDK1 PH domain [104]. This occurrence results in the inhibition of cell migration, 3D Matrigel invasion of breast cancer and melanoma cells and tumor dissemination in zebrafish xenotransplants.

## 6. Conclusions

While the function of PDK1 has been classically investigated within the context of the PI3K/Akt pathway, PDK1 plays role in several other pathways by phosphorylating and activating different kinases of the AGC family. PDK1 is an attractive target for cancer therapy due to its peculiar role in the regulation of cell motility, a fundamental process both in physiological and in pathological situations. PDK1 regulates cell locomotion through different mechanisms, such as activation of Akt [56,57,58], MRCKα [61], ROCK1 [60], β3 integrin [88] and PLCγ1 [64]. Moreover, a pivotal role for PDK1 in cancer progression has emerged in recent years [105]. Indeed, PDK1 has been shown to control growth and progression of several tumors: breast [106,107,108], prostate [109], pancreatic [99], gastric [100], colorectal [110,111]; ovarian [112], esophageal [113], gallbladder [114], acute myeloid leukemia [115] and melanoma [98,116].

Furthermore, results showing a reduced tumor occurrence in PTEN+/−, PDK1 hypomorphic mice, compared to PTEN+/− mice, strongly support PDK1 as important therapeutic target in cancer driven by alterations of the PI3K pathway [117]. Despite intensive investigation and promising preclinical data, clinical trials with inhibitors of this pathway have only partially met the initial expectations [118]; [119]. However, the use of PDK1 inhibitors could represent a valid alternative solution either as a single-agent approach or in combination with other inhibitors of the same pathway to overcome drug resistance.

In summary, PDK1 is a master kinase, able to control several physiological and pathological processes. Careful investigation has identified multiple ways by which PDK1 regulates cell migration and tumor growth and invasion. According to the experimental evidence accumulated so far, and reviewed here, PDK1 targeting could be effective to block cancer progression towards a more invasive and metastatic phenotype.

## Figures and Tables

**Table 1:** Different PDK1 conditional knockout models are listed in the table: the first column contains the tissues affected by the knockout and the promoter used for the Cre-recombinase expression; the second column contains a brief summary of the phenotype of the knockout; and the third column indicates the viability or lethality of knockout phenotype and the time when the lethality occurs.

Tissue (Promoter)	Phenotype	Viable/Lethal	References
Whole body	Lack of somites, forebrain and neural crest-derived tissue; vasculature not functional	Lethal E9.5	[23]
Cardiac muscles (MCK-Cre)	Heart failure; no activation of Akt and S6K. No activation of glycogen synthase after insulin stimulation; glucose uptake defects	Death between 5 and 11 weeks of age	[25,26]
Myocardium (αMHC-Cre)	Slow heart rate, decreased sodium current density	Death at 11 weeks of age	[27]
Myocardium (tamoxifen-inducible αMHC-Cre)	Cardiac dysfunction 1 week after Tamox; impaired responsiveness of βAR; increased apoptosis	Death at 5–15 weeks after tamoxifen	[28]
B cells (CD19-Cre)	Defective B cell development; increased apoptosis	Viable	[29]
Hematopoietic cells (Vav-Cre)	B cell development arrest; increased myeloid cell recruitment in lung and liver. Lack of Langerhans cells	Viable	[30,31]
T cells (CD4-Cre)	T cells activation and proliferation defects	Viable	[32,33]
Thymocytes (Lck-Cre)	No maturation of T cells	Viable	[34,35]
CD4 T cells/keratinocytes (OX40-Cre)	Inflammatory skin diseases	Viable	[36]
Keratinocytes (K14-Cre)	Thin and shiny epidermis; hypoplasia of vibrissae; deficient barrier function; asymmetric cell division defects	Death within several hours after birth	[37]
Neural precursors cells (Nestin-Cre)	Reduction in number of oligodendrocytes precursors cells during telencephalic development	Viable	[38]
Pancreas β cells (Rat insulin 2-Cre)	Alterate glucose homeostasis (diabetes); increased level of blood glucose and decreased level of insulin	Males die at 12.24 weeks of age	[39]
Pancreas progenitors (PDX1-Cre)	Pancreas hypoplasia; hyperglycemia; reduced number of endocrine and exocrine cells during development	Viable	[40]
Vascular endothelial cells (Tie2-Cre)	Growth retardation; hemorrhages; heart with abnormal morphology; defective vessels in yolk sac and in placenta; defective epithelial-mesenchymal transition	Lethal E11.5	[41]
